# Voxel-based analysis of grey and white matter degeneration in cervical spondylotic myelopathy

**DOI:** 10.1038/srep24636

**Published:** 2016-04-20

**Authors:** Patrick Grabher, Siawoosh Mohammadi, Aaron Trachsler, Susanne Friedl, Gergely David, Reto Sutter, Nikolaus Weiskopf, Alan J. Thompson, Armin Curt, Patrick Freund

**Affiliations:** 1 Spinal Cord Injury Center Balgrist, University Hospital Zurich, University of Zurich, Zurich, Switzerland; 2 Department of Systems Neuroscience, University Medical Center Hamburg-Eppendorf, Hamburg, Germany; 3 Wellcome Trust Centre for Neuroimaging, UCL Institute of Neurology, University College London, London, United Kingdom; 4 Department of Radiology, University Hospital Balgrist, Zurich, Switzerland; 5 Department of Neurophysics, Max Planck Institute for Human Cognitive and Brain Sciences, Leipzig, Germany; 6 Department of Brain Repair and Rehabilitation, UCL Institute of Neurology, University College London, London, United Kingdom

## Abstract

In this prospective study, we made an unbiased voxel-based analysis to investigate above-stenosis spinal degeneration and its relation to impairment in patients with cervical spondylotic myelopathy (CSM). Twenty patients and 18 controls were assessed with high-resolution MRI protocols above the level of stenosis. Cross-sectional areas of grey matter (GM), white matter (WM), and posterior columns (PC) were measured to determine atrophy. Diffusion indices assessed tract-specific integrity of PC and lateral corticospinal tracts (CST). Regression analysis was used to reveal relationships between MRI measures and clinical impairment. Patients showed mainly sensory impairment. Atrophy was prominent within the cervical WM (13.9%, p = 0.004), GM (7.2%, p = 0.043), and PC (16.1%, p = 0.005). Fractional anisotropy (FA) was reduced in the PC (−11.98%, p = 0.006) and lateral CST (−12.96%, p = 0.014). In addition, radial (+28.47%, p = 0.014), axial (+14.72%, p = 0.005), and mean (+16.50%, p = 0.001) diffusivities were increased in the PC. Light-touch score was associated with atrophy (R^2^ = 0.3559, p = 0.020) and FA (z score 3.74, p = 0.003) in the PC, as was functional independence and FA in the lateral CST (z score 3.68, p = 0.020). This study demonstrates voxel-based degeneration far above the stenosis at a level not directly affected by the compression and provides unbiased readouts of tract-specific changes that relate to impairment.

Cervical spondylotic myelopathy (CSM) is one of the most frequent spinal cord disorders leading to reduced independence and quality of life especially in the elderly^1^. Neurological impairment is mainly caused through chronic cord compression and deterioration of blood supply triggering degeneration of neural tissue in grey and white matter in the spinal cord at the level of stenosis^2^,^3^. The time course of the stenosis and its relation to the emergence of clinical impairment is unknown, as a moderate cord stenosis might be clinically silent for many years. Decompressive surgery is the therapeutic gold standard in patients with clinically relevant cord stenosis^4^, while a conservative approach is chosen in those patients with a radiologically less severe stenosis and only mild symptoms. Recently, novel experimental neuroprotective approaches improving outcome were investigated by blocking Fas-mediated apoptosis^2^ and the application of a benzothiazole anticonvulsant (i.e. Riluzole)^5^,^6^ and results were encouraging. Hence, there is a pressing need to detect and monitor early subclinical pathologic changes to the microstructure of the spinal cord, allowing interventions before those changes cause clinical impairments. However, neuroimaging biomarkers that can quantify disease progression at a stage before major irreversible damage occurs, monitor treatment effects (beyond decompression of the cord) and predict outcome are lacking^1^.

Recent developments in imaging and post-processing techniques have significantly advanced the accuracy and sensitivity of spinal cord neuroimaging. For example the advent of a common anatomical spinal cord template^7^, probabilistic tracts^7^, and optimization of post-processing techniques^8^ have now opened the avenue for unbiased voxel-based morphometry (VBM) analysis (i.e. without an a priori hypothesis) of volumetric as well as microstructural changes^7^,^8^. This enables the spatial localization of cord pathology and the determination of its relation with clinical impairment^9^,^10^.

In this study, we took advantage of high-resolution anatomical volumetric MRI to assess grey and white matter atrophy (i.e. Multiple Echo Data Image Combination (MEDIC)) and DTI to assess changes to the microstructural tissue properties. We hypothesised that chronic cord compression results in remote and spatially localized morphometric changes (i.e. atrophy) within cervical grey and white matter as well as tract-specific changes above the level of stenosis in CSM patients and that the degree of remote degeneration relates to clinical impairment.

## Results

### Subjects

In patients, impairment was assessed on the modified Japanese Orthopaedic Association (mJOA) score which identified ten patients suffering from mild (mJOA ≥ 15 [max. 18]), nine from moderate (mJOA = 12–14) and one from severe (mJOA < 12) CSM. The upper-extremity International Standards for Neurological Classification of SCI (ISNCSCI) scores were (mean ± SD) 27.70 ± 4.07 for light-touch (UELT) [max. 32], 27.30 ± 3.77 for pin-prick (UEPP) [max. 32], and 49.70 ± 0.57 for motor score (UEMS) [max. 50]. The spinal cord independence measure (SCIM) was 97.85 ± 4.04 [max. 100]. The total Graded Redefined Assessment of Strength, Sensibility and Prehension (GRASSP) score was 220.74 ± 12.32 [max. 232], and the sub-items were 95.16 ± 6.83 for strength [max. 100], 42.57 ± 6.50 for sensibility [max. 48], and 83.26 ± 1.45 for prehension [max. 84]. Individual scores are shown in Table 1.

### Conventional MRI measures at site of stenosis

The level of stenosis was at C5/C6 (n = 13) for the majority of patients, twice at C3/4, once at C4/C5, three times at C6/7, and once at C7/C8. In patients, the maximum spinal cord compression (MSCC) was 17.33 ± 7.21% and maximum canal compromise (MCC) 37.36 ± 8.58%. Nine patients showed a T2 signal hyperintensity with a signal change ratio of 1.58 ± 0.32. Individual data are shown in Table 1.

### Morphometric changes in the cervical cord above the site of stenosis

**Figure 1:**
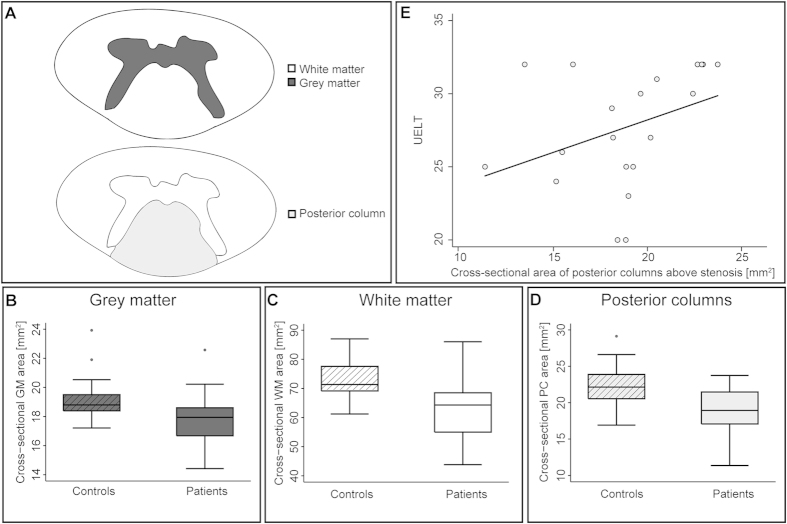
Morphometric changes in cervical spinal cord above compression site at C2/C3. (**A**) Schematic illustration of segmented cross-sectional cervical areas of grey matter, white matter, and posterior columns remote to compression. Significant reductions are shown for cross-sectional cervical cord area of grey matter (**B**), white matter (**C**), and posterior columns (**D**) in patients compared to controls. (**E**) Correlation between ISNCSCI upper extremity light-touch (UELT) score and cross-sectional area of the posterior columns (for illustrative purposes, not adjusted for age).

Volumetric as well as microstructural changes were observed in patients compared to controls above the level of stenosis at C2/C3. In patients, the cross-sectional cervical cord area was reduced by 12.8% (patients: 80.88 mm^2^, CI 75.59–86.17 mm^2^ vs. controls: 92.77 mm^2^, CI 88.88–96.67 mm^2^, p = 0.0032), GM area by 7.2% (patients: 17.80 mm^2^, CI 16.96–18.64 mm^2^ vs. controls: 19.18 mm^2^, CI 18.33–20.03 mm^2^, p = 0.0429), WM area by 13.9% (patients: 63.08 mm^2^, CI 58.42–67.75 mm^2^ vs. controls: 73.23 mm^2^, CI 69.88–76.57 mm^2^, p = 0.0041) and PC area by 16.1% (patients: 18.86 mm^2^, CI 17.30–20.41 mm^2^ vs. controls: 22.49 mm^2^, CI 21.06–23.92 mm^2^, p = 0.0051) (Fig. 1).

**Figure 2:**
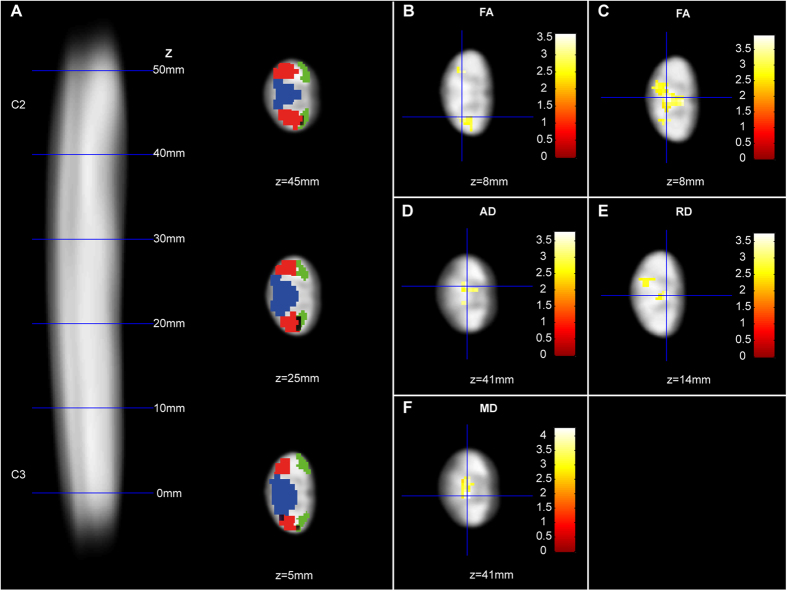
Tract-specific changes in microstructure above stenosis in patients with CSM compared to healthy controls. (**A**) Sagittal FA template of all subjects showing the cervical spinal cord at C2/C3 level and axial slices overlaid with regions of interest (lateral corticospinal tracts (CST) in red, posterior columns (PC) in blue, and spinothalamic tracts in green) for labeling of the anatomical position of the findings. (**B–F**) Overlay of statistical parametric maps (p < 0.01 uncorrected, shown for descriptive purposes, masked by the lateral CST and PC, respectively) revealing microstructural changes in patients compared to controls. In patients compared to controls, fractional anisotropy (FA) was reduced in the right lateral CST (**B**). In the PC, FA was reduced (**C**) and axial (**D**), radial (**E**), and mean diffusivity (**F**) increased. Colour bars indicate t-values. Axial slices are shown and their position is indicated by the z coordinate.

**Figure 3:**
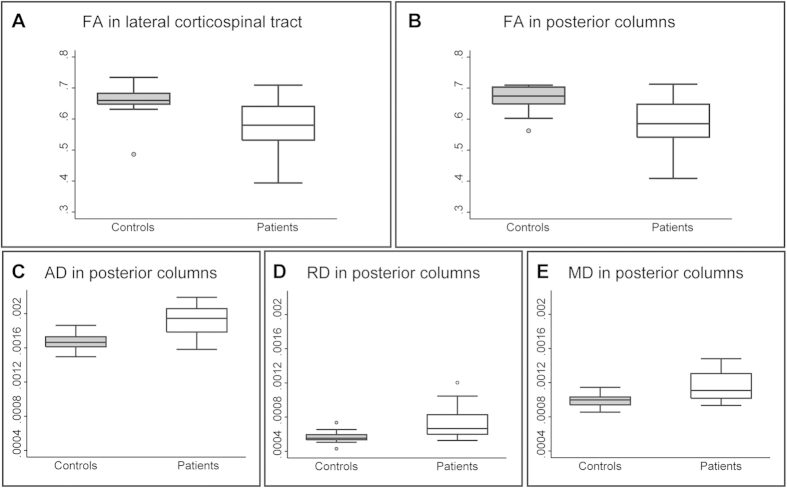
Quantification of tract-specific changes in microstructure above stenosis. In patients compared to controls, fractional anisotropy (FA) was reduced in the lateral CST (**A**) and PC (**B**), while AD (**C**), RD (**D**), and MD (**E**) were all increased in the PCs. Mean data were extracted from each significant cluster.

Voxel-based analysis of the cervical cord DTI revealed reductions in fractional anisotropy (FA) in the lateral CST (−12.96%, patients: 0.57, CI 0.53–0.61 vs. controls: 0.66, CI 0.63–0.68; x:4.5, y:−20.5, z:8.0, z score = 3.30, cluster extent = 144, p = 0.014, FWE corrected) and PC (−11.98%, patients: 0.59, CI 0.55–0.63 vs. controls: 0.67, CI 0.65–0.69; x:−2.0, y:−20.0, z:8.0, z score = 4.08, cluster extent = 174, p = 0.006, FWE corrected) while axial diffusivity (AD) (+14.72%, patients: 0.0019, CI 0.0019–0.0020 vs. controls: 0.0017, CI 0.0016–0.0017; x:−1.0, y:−18.5, z:41.0, z score = 3.91, cluster extent = 228, p = 0.005, FWE corrected), radial diffusivity (RD) (+28.47%, patients: 0.00073, CI 0.00064–0.00082 vs. controls: 0.00057, CI 0.00053-0.00060; x:1.0, y:−18.0, z:14.0, z score = 3.39, cluster extent = 203, p = 0.014, FWE corrected), and mean diffusivity (MD) (+16.50%, patients: 0.00073, CI 0.00064–0.00082 vs. controls: 0.00057, CI 0.00053–0.00060; x:2.0, y:−19.5, z:41.0, z score = 3.80, cluster extent = 379, p = 0.001, FWE corrected) were all increased in the PCs in patients when compared to controls (Figs 2 and 3).

### Clinical associations

**Figure 4:**
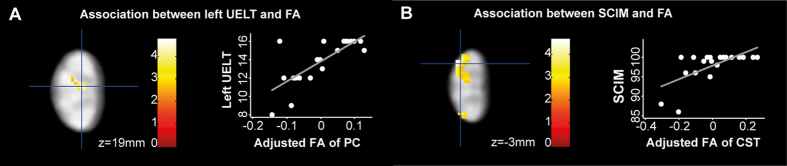
Associations between tract-specific microstructural readouts and clinical outcome above stenosis in patients with CSM. (**A**,**B**) Overlay of statistical parametric maps (p < 0.01 uncorrected, shown for descriptive purposes, masked by the lateral corticospinal tract (CST) and posterior columns (PC), respectively) revealing associations between DTI indices and clinical outcome (left panel) between cervical level C2 and C3 (see Fig. 2A for reference). Colour bars indicate t-values. The right panels illustrate the corresponding regression models extracted from the peak-voxel within the significant cluster. Diffusivity parameters of the correlations are mean centered and adjusted for age. In particularly, (**A**) lower fractional anisotropy (FA) in left PC was associated with worse left ISNCSCI upper extremity light-touch score (UELT) and (**B**) lower FA in the left lateral CST was associated with lower Spinal Cord Independence Measure (SCIM).

In patients, significant associations were seen between macro- and microstructural MRI readouts and impairment. In particular, reduced PC area was associated with lower ISNCSCI UELT scores (R^2^ = 0.3559, p = 0.020) (Fig. 1E). Furthermore, lower FA in the left PC was associated with lower left ISNCSCI UELT scores (x: 0.0, y:−18.5, z:19.0, z score = 3.74, p = 0.003, extent = 198, FWE corrected) (Fig. 4A). Lower FA in the left lateral CST was associated with lower SCIM score (x:−3.5, y:−21.5, z:−3.0, z score = 3.68, p = 0.020, extent = 135, FWE corrected) (Fig. 4B).

## Discussion

This study in patients with CSM shows, next to conventional MRI measures (e.g. MSCC, MCC, signal change ratio)^11^, for the first time remote and spatially localized morphological and microstructural changes of the spinal cord within the grey and white matter above the level of stenosis. In particular, there was grey and white matter atrophy, which was paralleled by tract-specific microstructural changes in major spinal pathways. Crucially, the degree of spinal cord pathology correlated with measures of clinical impairment of posterior column and pyramidal dysfunction. Thus, unbiased voxel-based analysis revealing spatially localized cord pathology provides complementary pathophysiological insights into rostral degenerative changes beyond the routine clinical work up (i.e. clinical examination, electrophysiology and radiology) of patients suffering from CSM.

Volumetric MRI provides information on (macro-structural) general tissue loss (e.g. atrophy), whereas DTI provides information on fiber orientation and tissue structure and thus is sensitive to microstructural tissue properties. While decreased FA has been associated with both axonal count^12^ and myelin content^13^, increased RD and decreased AD have been associated with demyelination^14^ and axonal degeneration^15^, respectively.

This study found marked tissue loss (i.e. atrophy) of white and grey matter and tract-specific microstructural changes within major ascending and descending spinal pathways above the site of cord compression. The degree of atrophy above the site of stenosis was greater in the white matter (−13.9%) and less strong in grey matter (−7.2%). Changes in the spinal cord microvasculature^3^ and distribution of blood flow^6^,^16^, disruption of blood spinal cord barrier^3^, and neuroinflammation^2^,^3^ could make motoneurons above the compression site vulnerable to degeneration and apoptosis and be potential mechanisms for grey matter atrophy.

Moreover, we found tract-specific decreased FA within the lateral CST and PC, and increased RD and MD in the PC as markers of axonal degeneration and demyelination^12^,^13^,^14^,^15^,^17^,^18^ next to the reported white matter atrophy. These findings are coherent with other studies^19^,^20^,^21^. We found AD to be increased within the PC and these results are consistent with regards to the change of AD as shown in previous studies of CSM^20^,^22^,^23^. This might be due to elevated fiber tract density due to compression and loss of surrounding structure, as reported at the lesion site in patients with CSM^24^, but also caudal and rostral to the compression site^25^, that manifests over time. AD has been shown to behave bi-directionally over time in patients with optic neuritis and in experimental spinal contusion studies^26^,^27^. In the latter AD decreased rapidly in rostrocaudal direction, but increased over time at the lesion site^27^. Nevertheless, in our study we report that the difference between patients with CSM and healthy controls in RD is nearly double as high as in AD which is suggestive that demyelination rather than axonal degeneration is occurring above the level of stenosis. It would be interesting to investigate our reported findings in other spinal cord pathologies at the same cervical level. Our conjoint analysis of macro- as well as microstructural changes are of interest because they suggest that a combination of pathological processes at the microstructural level including long distance retro- as well as anterograde fiber degeneration (i.e. axonal loss, demyelination) and potentially inflammation^3^ and to a lesser extent neuronal changes (i.e. apoptosis^2^,^3^ and anterior horn cell loss^6^,^28^) occur several segments above the level of stenosis.

The magnitude of remote changes in terms of atrophy and microstructural changes resembles those observed in traumatic spinal cord injury (SCI)^18^,^29^,^30^,^31^,^32^,^33^. Although based on two very different underlying aetiologies (abrupt onset in traumatic SCI, slowly developing symptoms in CSM), CSM and SCI clearly result in focal damage to the spinal cord with a combination of alpha-motoneuron damage (lesion of the central grey) as well as demyelination and axonal damage of long projecting spinal nerve fibre tracts (white matter damage)^1^,^34^. Herein we provide converging evidence that both aetiologies show remote cord pathology to a similar extent despite their striking difference in levels of disability. Given the marked degenerative changes above the site of stenosis, we anticipate atrophy and microstructural changes also to occur caudal to the site of stenosis contributing to the frequently seen gait disturbance^1^.

Associations between clinical outcomes and cord pathology at the level of cord damage have been reported previously^11^,^24^,^35^. Here we show that the structural changes above the site of stenosis carried within specific tracts are related to measures of functional independence (i.e. SCIM) within the lateral CST and sensory deficits (i.e. UELT) within the PC. The latter finding is striking as it suggests that greater structural perturbations, most probably corresponding to a combination of pathologic processes, contribute to sensory impairment. In contrast, despite signs of marked structural integrity changes of the lateral CST, most patients had normal or only mild pyramidal impairment. In other words, good clinical scores but marked structural changes may portend risk for a progressive functional decline.

We note the following considerations and limitations of this study. Firstly, the average age difference between gender matched patients and healthy controls was 7.6 years though this difference was not statistically significant. In all models, we included age as a regressor of no interest to reduce effects of age on our results. Secondly, the segmentation of the cervical cord into grey matter and posterior columns was performed manually, but the inter-, and intra-rater coefficients of variation for the cervical cord were similar to those published^36^. Finally, VBM-style analysis of spinal cord DTI is in its infancy^7^,^9^,^10^ and as with the early days of exploring brain DTI, there are limitations associated with this method. For example, spatial mis-registration can lead to false positive or false negative results and thus reduce the reliability of this emerging post-processing method for spinal cord DTI^8^. To reduce these, we performed an additional registration step to reduce residual mis-registrations that were not sufficiently removed by our proposed automated processing method. In future work, advanced spatial normalization methods such as Dartel^37^ could, if adopted to the spinal cord, help to improve registration quality and thus further advance VBM-style analysis of DTI in the spinal cord.

Another important issue of spinal cord DTI is the fact that instrumental and physiological noise artefacts are larger in the spinal cord than in the brain. This can reduce the reproducibility of spinal cord DTI metrics. To minimize artefacts, we applied sophisticated imaging and preprocessing methods. In particular, we reduced fold-over artefacts due to incomplete outer-volume suppression using 50% phase oversampling and by adjusting the amplitude of the saturation pulse manually for each subject. To minimize physiological noise artefacts, all patients were equipped with a stiff neck to reduce motion in the z-direction during scanning^36^. Further, we used a pulse-triggered sequence and optimized pre-processing techniques^38^ to mitigate the adverse effect of subject motion and other physiology-related artefacts (e.g. heart-beat or breathing). To assess the amount of variability due to residual artefacts, we tested the scan-rescan reliability of our data using a jackknife analysis as described in Mohammadi *et al.* (2013) (data analyses not shown). We found that the intra-subject variability (averaged across subjects) was about Δ-FA = 0.02, which is 3% when compared to the reported averaged FA values in the spinal cord (0.02/0.66*100 = 3%). This is smaller than the reported inter-subject variability of Δ-FA = 0.05 (reported in the Results section), which corresponds to 8% variability (0.05/0.66*100% = 8%). Thus, we expect that the scan-rescan variability will not strongly alter the reported effects observed between controls and patients. However, we cannot exclude that the reported high inter-subject variability might be due to the fact that data quality varies between subjects, e.g. variation in motion artefacts between subjects.

To further improve the reproducibility of spinal cord DTI, optimizations at the level of the sequence (e.g. navigators that help discarding poor-quality scans as used in segmented EPI^39^) and post-processing techniques (e.g. denoising) as used in brain DTI^40^ should be considered.

## Conclusion

Atrophy and tract-specific degeneration of the cervical cord can be detected by means of high-resolution MRI and expands far beyond focal cord damage as assessed by conventional MRI (e.g. MCC, MSCC, and signal change ratio). Importantly, these remote tract-specific changes are related to the clinical presentation and may be applicable as surrogate markers to assess treatment effects (in surgical and drug based interventions) complimentary to clinical outcome measures. Thus, MRI findings above and potentially below the site of stenosis could disclose subclinical sensory and motor impairments of upper and lower limb impairment and complement the routine clinical and electrophysiological assessment^41^ in individuals with CSM.

## Material and Methods

### Subjects and study design

Twenty patients (six women) (Table 1) referred for a neurological evaluation due to CSM with a mean age of 52.0 ± 14.5 (SD) years and 18 gender-matched controls (six women) with a mean age of 44.4 ± 9.7 (SD) years were consecutively recruited for this study between July 2012 and September 2014 in the outpatient clinic at the University Hospital Balgrist, Switzerland. The mean age between both groups was statistically not different (Mann-Whitney U test: z = −1.61, p = 0.1075). The participants fulfilled the following inclusion criteria: no other neurological or mental disorders affecting clinical outcome, age between 18 to 70 years, no MRI contradictions, and no pregnancy. The study protocols were in accordance with the Declaration of Helsinki and were approved by the local Ethics Committee of Zurich, the *‘Kantonale Ethikkommission Zurich’* (ref. number: EK-2012-0343). All participants provided written informed consent.

### Clinical examination

All patients received a comprehensive clinical examination including the modified Japanese Orthopedic Association (mJOA) scale^42^ [max. 18 points], and additional outcome measures such as the ISNCSCI protocol^43^ for UEMS (e.g. pyramidal dysfunction) [max. 50 points], UELT (e.g. posterior column dysfunction) [max. 32 points], and UEPP (e.g. spinothalamic dysfunction) [max. 32 points], SCIM [max. 100 points])^44^, and the GRASSP protocol [max. 232 points] as ancillary outcome measure sensitive for upper extremities in CSM^45^ (sub-item for strength [max. 100 points], sensibility [max. 48 points], and prehension [max. 84 points])^46^. Based on the mJOA, CSM severity was identified as mild (mJOA ≥ 15), moderate (mJOA = 12–14), or severe (mJOA < 12)^47^.

### Image acquisition

All participants were scanned on a 3T Skyra MRI scanner (Siemens Healthcare, Erlangen, Germany) equipped with a 16-channel radio-frequency (RF) receive head and neck coil and RF body transmit coil. All participants wore an MRI-compatible stiff neck (Laerdal Medicals, Stavanger, Norway) to minimize motion artefacts and were carefully positioned by the radiographers to acquire the data from the same position and to obtain high reproducibility between all participants.

A 2D sagittal T2-weighted turbo spin-echo sequence was used for anatomical assessment of cervical spinal cord covering the levels of stenosis. Following parameters were applied to acquire 20 slices within 2 minutes and 2 seconds: slice thickness 2.5 mm with 10% inter-slice gap, field of view (FOV) of 220 × 220 mm^2^, matrix size of 384 × 384, time of repetition (TR) of 3760 ms, time of echo (TE) of 87 ms, flip angle α = 160°, and readout bandwidth of 260 Hz per pixel.

A 3D high-resolution optimized T2*-weighted multi-echo sequence (multiple echo data image combination; MEDIC)^48^ was applied to acquire five high-resolution axial 3D volumes of the cervical cord above the stenosis at C2/C3 level. Each volume consisted of twenty partitions and was obtained with a resolution of 0.25 × 0.25 × 2.50 mm^3^ within 2 minutes and 8 seconds for each of the five volumes. Following parameters were applied: FOV of 162 × 192 mm^2^, matrix size of 648 × 768, TR of 44 ms, TE of 19 ms, flip angle α = 11°, and readout bandwidth of 260 Hz per pixel.

The acquisition of DTI data used a cardiac-gated monopolar sequence (based on finger pulse oximetry)^49^ and the following parameters: 30 diffusion-weighted (DW) images (b = 500 s/mm^2^), six T2- weighted images without diffusion weighting (b  = 0), 5 mm slice thickness, with 10% inter-slice gap, 10 slices perpendicularly oriented to the spine, 5/8 Partial-Fourier Imaging in phase-encoding direction, phase oversampling 50%, and a cardiac trigger delay of 200 ms, 176 × 40 acquisition matrix, FOV of 133 × 30 mm^2^, 0.8 × 0.8 mm^2^ in-plane resolutions, TE of 73 ms, and TR of 350 ms. The gated data were acquired in blocks of two slices per cardiac cycle. The minimal time between successive triggers was 1800 ms. Reduced field of view was achieved by outer-volume suppression^49^ using two spatial saturation pulses placed anterior and posterior to the spinal cord, along the phase-encoding direction^50^. To reduce fold-over artefacts due to insufficient outer volume suppression, the amplitude of the saturation pulse was adjusted for each subject individually. Each DTI dataset was acquired four times at the same C2/C3 position in the cervical spinal cord, resulting in 144 images for each subject. Altogether, this resulted in a total acquisition time of about 6.2 minutes (as estimated by the sequence simulator), but could be longer depending on the participant’s heart rate.

### Processing of sagittal T2-weighted data of cervical spine

We used JIM 6.0 (Xynapse Systems, Aldwincle, UK) to calculate the maximum canal compromise (MCC) and maximum spinal cord compression (MSCC) using the midsagittal slice^11^,^51^. We calculated the signal change ratio from the region of hyperintensity or, if not applicable, from the level of greatest cord compression against an average reference on the spinal cord at C7/T1 and C2 using the ratio proposed by Nouri *et al.*^11^.

### Processing of high-resolution structural data above the level of stenosis

We used a symmetric diffeomorphic algorithm^52^, that is embedded in SPM12 (Wellcome Trust Centre for Neuroimaging, University College London, London, UK), to register the five 3D MEDIC volumes in each subject in order to account for non-rigid body motion effects and create an average volume for a better signal to noise ratio (SNR). Jim 6.0 was then used to merge the adjacent partitions to get 10 contiguous slices (to increase SNR) and to semi-automatically segment the cross-sectional cervical cord area using an active-surface model after setting a marker in the center of the cord in each of the 10 contiguous slices^53^. The GM and PC were extracted manually in each slice. WM was estimated by subtracting the GM from cervical cord area. The segmentation was performed by two independent investigators (PG and AT) and was reevaluated a second time with a gap of at least two weeks in between. Coefficient of variations (=standard deviation divided by mean) for cervical cord area, WM, GM, and PC were 0.6%, 1.9%, 6.9%, and 3.4% for inter-rater and 0.9%, 1.3%, 4.4%, and 1.4% for intra-rater variations, respectively.

### Pre-processing and estimation of DTI data

The DTI data were interpolated to a higher in-plane resolution of 0.4 × 0.4 mm^2^. Then, the data were corrected for motion and eddy current artefacts using a novel constrained 3D-affine registration^54^ that corrected for selected rigid-body subject motion and linear eddy current distortion parameters (details on linear eddy currents can be found in^55^). We restricted the 3D-affine registration to correct only for in-plane x- and y-translation (mainly caused by subject motion and eddy currents) as well as for in-plane scaling and in-plane shearing along the y-direction (both mainly caused by eddy currents). We did not correct for rotation and through-plane shearing effects, which were less pronounced and less robust to estimate. To correct for residual artefacts associated with subject motion and physiological noise^38^, we used robust fitting as implemented in the ACID toolbox (www.diffusiontools.com). Combining eddy current and motion correction with the robust tensor fitting approach has been shown to be particularly efficient to reduce intra-scan variability of DTI metrics^56^.

### Post-processing and spatial normalization of DTI index maps

First, the in-plane field-of-view was chopped to 24 × 24 mm^2^ for each DTI dataset to exclude non-spine tissue. Then, the following pathological relevant DTI index maps^9^,^13^,^29^ were calculated: FA, RD, AD, and MD. These DTI index maps were spatial normalized into the space of the AMU white and grey matter probabilistic tracts embedded within the MNI–Poly–AMU template^7^ using the FA voxel-based statistics (FA-VBS) toolbox^8^ with refined spatial normalization parameters, which specifically accounted for the anatomy of the spinal cord, i.e. the degree of freedom of the spatial transformation was reduced along the head-foot direction. To use optimal and complementary contrast information, we used the MD and FA contrast to drive the spatial normalization. Then, we performed additional manual slice-by-slice registration (shift and scaling along the phase-encoding direction) to refine to accuracy of the registration. Finally, all DTI index maps were smoothed using a Gaussian kernel with 0.5 × 0.5 × 5 mm^3^ with full-width at half maximum (FWHM).

### Statistical analysis

Stata 13 (StataCorp LP, Texas, USA) was used for statistical analysis of clinical and morphometric data. We used Mann-Whitney U tests to investigate age differences between patients and controls. We used ANCOVA with age as covariate of no interest to assess morphometric differences between patients and controls in cross-sectional cervical cord area, GM, WM, and PC. Linear regression models were used to determine associations between bilateral clinical outcome measures for mJOA, UELT, UEPP, GRASSP sensibility, strength, and prehension, SCIM, MSCC, MCC, and signal change ratio and cross-sectional cervical cord area and between UELT and GRASSP sensibility and PC. Bilateral outcome measures were used because there is not landmark to segment left and right cross-sectional cervical cord measures separately. Age was included and treated as covariate of no interest to adjust for possible confounds of age in every statistical test. The level of significance was defined as α = 0.050.

SPM12 was used for voxel-based statistics of the different DTI indices. To this end, we investigated the averaged DTI indices across the cross-section of the cervical cord (C2/C3) and verified the tract-specific location using the white matter spinal cord atlas that is embedded in the Spinal Cord Toolbox^57^ for the PC, lateral CST, and spinothalamic tracts. We used ANCOVA to investigate cross-sectional differences in DTI indices (e.g. FA, AD, RD, and MD) between patients and controls and adjusted for age within the groups.

Linear regression models were constructed for associations between unilateral clinical outcome measures for UELT, UEPP, GRASSP sensibility, strength, and prehension and DTI indices (except for mJOA, SCIM, MSCC, MCC, and signal change ratio as purely bilateral outcome measures). Age was included as a covariate of no interest in all linear regression models to adjust for possible confounds of age. All statistical parametric maps were initially thresholded with a cluster defining threshold of uncorrected p < 0.01. Clusters surpassing a cluster threshold of p = 0.05, corrected for family-wise error based on Gaussian Random Field theory, are reported^58^.

## Additional Information

**How to cite this article**: Grabher, P. *et al.* Voxel-based analysis of grey and white matter degeneration in cervical spondylotic myelopathy. *Sci. Rep.* **6**, 24636; doi: 10.1038/srep24636 (2016).

## Figures and Tables

**Table 1:** Clinical and behavioural data of 20 patients with cervical spondylotic myelopathy.

	Gender	Age	Level of stenosis	mJOA	UEMS	UELT	UEPP	SCIM	GRASSP Strength	GRASSP Sensibility	GRASSP Prehension	MSCC (%)	MCC (%)	T2 Hyperintensity	Signal Change Ratio
1	m	36	C5/6^a^	12	48	20	25	99	74	37	84	8.35	39.14	No	0.93
2	m	50	C5/6^a^	16	49	25	26	100	100	47	84	23.85	48.37	No	1.03
3	m	39	C3/4^a^	13	50	25	23	100	98	43	84	24.38	40.83	No	1.00
4	f	66	C5/6	12	49	32	32	96	95	37	84	14.14	36.67	Yes	1.33
5	m	69	C5/6^a^	17	50	32	32	100	99	44	84	16.31	39.59	No	1.12
6	m	68	C6/7^a^	17	50	32	32	100	NT	NT	NT	26.12	49.81	Yes	1.77
7	m	39	C5/6	16	50	32	25	100	100	46	84	29.33	52.72	No	1.13
8	m	34	C5/6	14	50	30	30	100	100	47	84	23.73	37.29	No	0.99
9	f	31	C5/6	16	50	27	27	100	100	48	84	11.40	34.04	Yes	1.55
10	f	47	C5/6^a^	16	50	31	31	100	100	48	84	12.84	39.94	Yes	1.35
11	m	63	C4/5^a^	12	50	24	24	95	94	41	84	13.82	30.67	Yes	1.81
12	m	74	C6/7^a^	14	50	32	32	88	89	42	84	22.62	43.01	Yes	1.34
13	m	32	C5/6^a^	16	50	20	20	100	100	48	84	1.13	27.14	None	1.03
14	m	66	C5/6	9	50	25	26	86	99	34	82	18.17	26.44	Yes	2.27
15	m	51	C5/6	15	50	27	27	96	92	44	81	17.07	36.32	No	1.00
16	f	53	C5/6	16	50	32	30	100	100	46	84	18.76	49.98	Yes	1.46
17	m	72	C7/T1^a^	14	50	26	22	100	98	41	83	25.82	32.07	No	1.05
18	f	37	C3/4^a^	14	49	30	30	99	93	46	79	16.03	23.00	Yes	1.31
19	f	58	C5/6^a^	16	49	29	29	100	93	48	84	15.64	34.96	No	1.00
20	m	55	C6/7	12	50	23	23	98	84	22	81	7.15	25.25	No	1.06

mJOA = modified Japanese Orthopaedic Association [max. 18 points]. UEMS = Upper extremity motor score [max. 50 points]. UELT = Upper extremity light-touch [max. 32 points]. UEPP = Upper extremity pin-prick [max. 32 points]. SCIM = Spinal Cord Independence Measure [max. 100 points]. GRASSP = Graded Redefined Assessment of Strength, Sensibility and Prehension [strength: max. 100 points, sensibility: max. 48 points, prehension: max. 84 points]. MSCC = Maximum Spinal Cord Compression. MCC = Maximum Canal Compromise. NT = Not tested. ^a^Multisegmental degeneration of cervical spine.
